# Immunomodulatory effects of canine mesenchymal stem cells in an experimental atopic dermatitis model

**DOI:** 10.3389/fvets.2023.1201382

**Published:** 2023-07-13

**Authors:** Seok-Jin Kang, Na-Yeon Gu, Jeong Su Byeon, Bang-Hun Hyun, Jienny Lee, Dong-Kun Yang

**Affiliations:** ^1^Viral Diseases Research Division, Animal and Plant Quarantine Agency, Gimcheon, Republic of Korea; ^2^Division of Regenerative Medicine Safety Management, Department of Chronic Disease Convergence Research, Korea National Institute of Health, Korea Disease Control and Prevention Agency, Cheongju, Republic of Korea

**Keywords:** canine, atopic dermatitis, dermatophagoides farina extract, MSCs, immunomodulatory effect

## Abstract

Mesenchymal stem cells (MSCs) have the potential to differentiate into multi-lineage cells, suggesting their future applicability in regenerative medicine and biotechnology. The immunomodulatory properties of MSCs make them a promising replacement therapy in various fields of animal research including in canine atopic dermatitis (AD), a skin disease with 10–15% prevalence. We investigated the immunomodulatory effects of MSCs in an experimental canine AD model induced by *Dermatophagoides farinae* extract ointment. Canine adipose tissue-derived MSCs (cAT-MSCs) were differentiated into mesodermal cell lineages at the third passage. Alterations in immunomodulatory factors in control, AD, and MSC-treated AD groups were evaluated using flow cytometric analysis, enzyme-linked immunosorbent assay, and quantitative reverse transcription PCR. In the MSC-treated AD group, the number of eosinophils decreased, and the number of regulatory T cells (Tregs) increased compared to those in the AD group. In addition, the immunoglobulin E (IgE) and prostaglandin E_2_ levels were reduced in the MSC-treated AD group compared to those in the AD group. Furthermore, the filaggrin, vascular endothelial growth factor, and interleukin-5 gene expression levels were relatively higher in the MSC-treated AD group than in the AD group, however, not significantly. cAT-MSCs exerted immunomodulatory effects in an AD canine model via a rebalancing of type-1 and -2 T helper cells that correlated with increased levels of Tregs, IgE, and various cytokines.

## 1. Introduction

Atopic dermatitis (AD) is a chronic and recurrent inflammatory skin disease involving skin barrier dysfunction and aberrant immune responses to allergens in genetically susceptible individuals (1, 2). AD shares many clinical and pathogenic features in humans and canines. Human AD is associated with an imbalance in type-1 and type-2T helper (Th) cells and an altered state of immunosuppression, which is induced by regulatory T cells (Tregs) (3, 4). The latter is a special T-lymphocyte subset and includes both naturally occurring thymus-derived and peripherally-induced Tregs (5, 6). Tregs exhibit immunoregulatory properties that play an important role in immunological homeostasis maintenance (7, 8). Thus, irregularities in the number or function of Tregs are thought to contribute to allergic disease pathogenesis (3, 9, 10). The number of circulating Tregs is altered in patients with AD compared with those in healthy humans (9, 11).

Atopic dermatitis is one of the most common canine skin diseases. It is defined as a genetically predisposed inflammatory and pruritic allergic skin disease with characteristic clinical features (12). These features include immunoglobulin E (IgE) accumulation against environmental allergens (13), imperfect epidermal barrier function, allergen processing by epidermal Langerhans cells, T-lymphocyte cytokine response imbalance, increased cutaneous mast cell release, and heightened sensitivity to secondary bacterial and yeast infections (14). In addition, given the similarities in human and canine immune systems, Tregs are likely to play a critical role in canine AD; however, very little is known about such a role.

Mesenchymal stem cells (MSCs) are multipotent cells that can differentiate into various cell lineages and have implications for cell-based research, including regenerative medicine and reproductive biotechnology. Canine adipose tissue-derived MSCs (cAT-MSCs) express genes associated with multipotency whereas their differentiated progeny express appropriate lineage-specific genes. Canine-derived MSCs have been established from various tissues, such as adipose tissue (15), the umbilical cord (16), umbilical cord vein (17), umbilical cord blood (18), and bone marrow (19). Canine MSCs can be used as alternative biological materials for the treatment of various diseases, such as osteoarthritis (20), liver disease (21), liver fibrosis (22, 23), and kidney injury in animal models (24). Moreover, MSCs affect immune responses, such as T cell development (25), B cell activity (26), and NK cell proliferation (27). MSCs secrete numerous cytokines, chemokines, growth factors, and extracellular vesicles. These factors act as immunomodulators in signal transduction pathway activation and are involved in various physiological and pathological processes. Thus, canine MSCs could be considered as a novel strategy to treat canine AD. To address these expectations, this study examined cAT-MSC immunomodulatory effects on Tregs, IgE, and cytokine levels in dogs treated with *Dermatophagoides farinae* extract (DFE).

## 2. Materials and methods

### 2.1. cAT-MSC isolation

Canine abdominal adipose tissues were collected, washed twice with phosphate-buffered saline (PBS; Thermo Fisher Scientific, Waltham, MA, United States) containing 1× P/S (100 units/mL penicillin, 100 μg/mL streptomycin; Thermo Fisher Scientific), diced with scissors, and incubated in Dulbecco’s PBS containing 0.1% collagenase type I (Thermo Fisher Scientific) for 1 h at 37°C. The tissue suspension was filtered using a 100 μm cell strainer and centrifuged at 400 × *g* for 5 min, whereafter the supernatant was discarded. Dulbecco’s Modified Eagle’s Medium (Thermo Fisher Scientific) containing 10% fetal bovine serum (FBS; Thermo Fisher Scientific) and 1× P/S were added to the centrifuged pellet followed by thorough mixing, and the cells were dispensed into a cell culture flask and cultured in an incubator at 37°C and 5% CO_2_. The following day, the medium was changed, and the cells were treated with 0.25% trypsin-ethylenediaminetetraacetic acid (EDTA) when 80% confluent prior to being cultured at the subpassage.

### 2.2. cAT-MSC *in vitro* differentiation

To confirm the cAT-MSC mesodermal differentiation ability, the media for adipogenesis, chondrogenesis, and osteogenesis induction was replaced over 2–3 days, and differentiation was induced over 21 days (Table 1). The differentiated cells were washed twice with PBS, fixed with 4% paraformaldehyde for 10 min, and then rewashed with PBS. To evaluate adipogenic differentiation, the fixed cells were stained with Oil Red O staining kit (IHC World, Ellicott City, MD, United States), with red lipid vacuoles accumulating in the differentiated cells. After chondrogenic differentiation, the cells were fixed and stained with Alcian Blue (IHC World) to detect the presence of glycosaminoglycans. Following osteogenic differentiation, the presence of extracellular calcium was confirmed using an Alizarin Red staining kit (IHC World).

**Table 1:** Differentiation media components.

Adipogenesis	Dexamethasone	1 μM
	3-isobutyl-1-methylxanthine	500 μM
	Indomethacin	100 μM
	Insulin	10 μg/mL
Chondrogenesis	Dexamethasone	0.1 μM
	Ascorbic acid 2-phosphate	100 μg/mL
	TGF-β	10 ng/mL
Osteogenesis	Dexamethasone	0.1 μM
	Ascorbic acid 2-phosphate	50 μg/mL
	β-glycerophosphate	10 mM

### 2.3. Establishment of animals with experimental AD and peripheral blood mononuclear cell isolation

**Figure 1:**
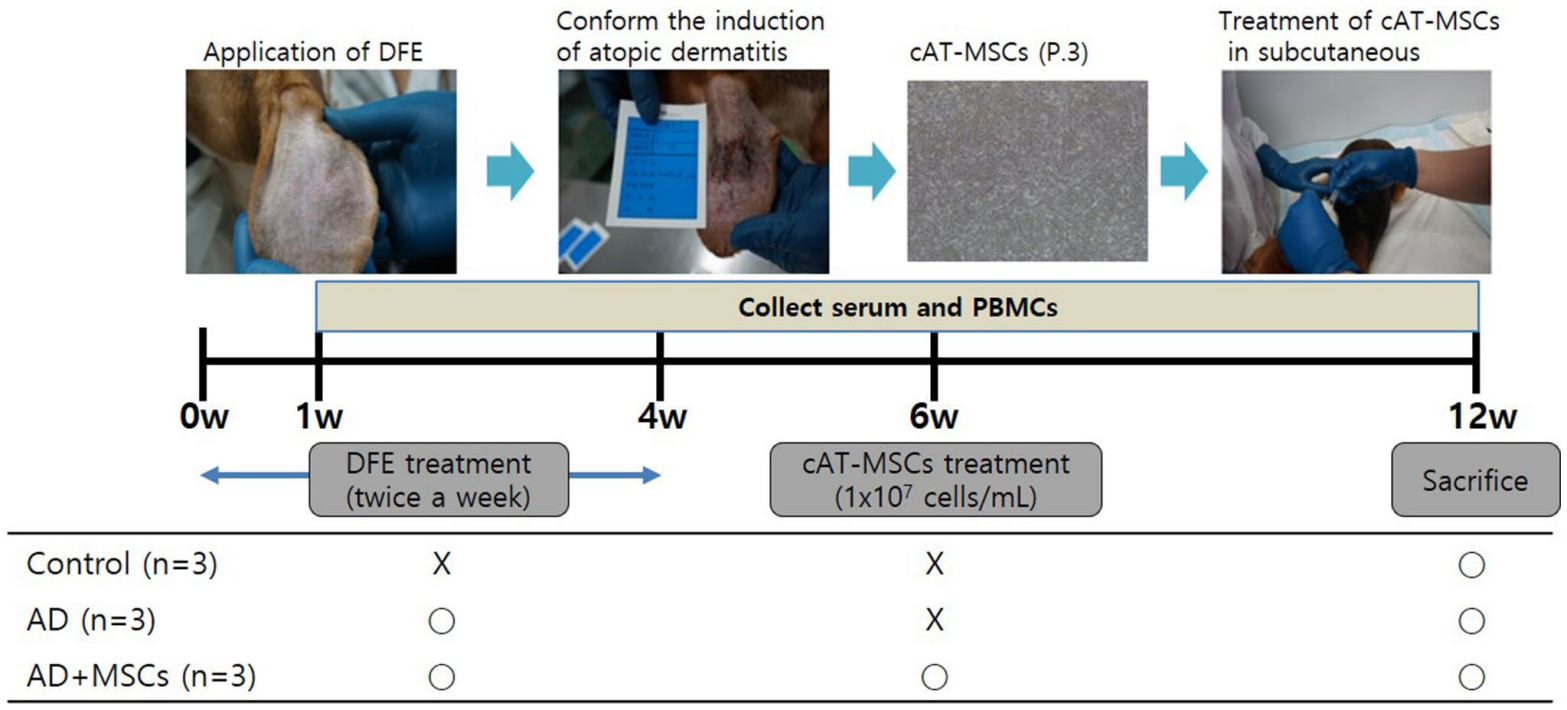
Experimental design. Nine dogs were randomly assigned to three groups: control (*n* = 3), AD (*n* = 3), and AD + MSCs (*n* = 3). Canine atopic dermatitis was induced by DFE ointment application over 4 weeks, and cAT-MSCs (1 × 10^7^ cells/mL) were subcutaneously injected into dogs after 6 weeks. Dogs were euthanized after 12 weeks. Serum and PBMCs were collected weekly during the experimental period. AD, atopic dermatitis; cAT-MSC, canine adipose tissue-derived mesenchymal stem cell; DFE, *Dermatophagoides farinae* extract; MSC, mesenchymal stem cell; and PBMC, peripheral blood mononuclear cell.

The dogs used in this study were purpose-bred laboratory beagles obtained from Korean animal medical science institute (KAMSI) (Incheon, Korea). All experiments were approved by and followed the guidelines of the Institute of Laboratory Animal Resources (15-KE-047). As shown in Figure 1, nine beagles were randomly divided into three groups, control (*n* = 3), AD (*n* = 3), and MSC-treated AD (AD + MSCs, *n* = 3). Prior to AD induction, the earlobe surfaces were shaved with a clipper, with any remaining hair removed using surgical tape. Thereafter, 100 mg of DFE ointment (Biostir Inc., Hiroshima, Japan) was applied twice a week for 4 weeks (i.e., a total of eight times) to each ear. The cAT-MSCs (1 × 10^7^ cells/ mL) were injected at week 6, and the dogs were euthanized at week 12.

After AD induction, blood samples were collected weekly via venipuncture of the jugular vein without anesthesia into 10-mL sodium heparin tubes (BD Vacutainer Systems, Franklin Lakes, NJ, United States). PBMCs were separated from the samples via density gradient centrifugation using Ficoll Paque (density = 1.077; GE Healthcare Bio-Sciences, Uppsala, Sweden). Briefly, whole blood samples were diluted 1:1 in PBS, layered onto Ficoll Paque, and centrifuged at 1,800 rpm for 20 min. The PBMCs were collected, washed twice with PBS, and resuspended in Roswell Park Memorial Institute 1640 medium (Thermo Fisher Scientific) supplemented with 1× P/S and 10% FBS.

### 2.4. Enzyme-linked immunosorbent assay

Immunoglobulin E and prostaglandin E_2_ (PGE2) levels were determined using an IgE Dog ELISA kit (E40-125, Bethyl laboratories, Inc., Montgomery, TX, United States) and PGE2 parameter assay kit (SKGE004B, R&D systems, Minneapolis, MN, United States), which were used according to the manufacturers’ protocols. Serum was diluted 3-fold with each diluent and then applied in triplicate (100 μL) to a 96-well plate for analysis. The optical density (OD) of each standard, sample, and control was determined at 450 nm in triplicate, with the average OD used for the analyses. The mean OD value of each analyte was subtracted from that of the blank control to construct a standard curve. The concentrations of the samples were determined using the corresponding mean absorbance from the standard curve. All samples were tested on the same day.

### 2.5. Flow cytometric analysis

The cAT-MSCs were detached from culture plates using 0.25% trypsin/EDTA and washed with PBS. To identify stem cell surface markers, the cells (1 × 10^5^) were labeled with the following antibodies for 1 h: mouse anti-dog cluster of differentiation CD34 (559369, BD Bioscience, United States), mouse anti-dog CD44 (MAB5549, R&D system), and rat anti-dog CD90 (12-5900, eBioscience, United States). The labeled cells were washed twice with PBS and analyzed with a FACSCalibur flow cytometer (Becton Dickinson, United States), using Cell Quest Pro software (Becton Dickinson) for data analysis.

For cell surface staining, PBMCs were collected, resuspended at 2 × 10^6^ cells per 100 μL, and washed twice before blocking in cell-staining buffer (BioLegend, United States). Briefly, PBMCs were incubated with rat anti-dog CD4 (MCA1038F, AbD Serotec, United States), mouse anti-dog CD25 (12-0250, eBioscience), or isotype control antibodies for 20 min in the dark. The cells were then washed with cell-staining buffer and fixed with fixation/permeabilization solution (00-5223, eBioscience) for 30 min. For intracellular staining, cells were washed twice with 1 × permeabilization solution (eBioscience). After washing, the cells were incubated with rat anti-mouse/rat Foxp3 (12-5773, eBioscience) or isotype control for 30 min in the dark. The PBMCs were washed twice, resuspended in staining buffer, and analyzed using a FACS Calibur flow cytometer. CD4/CD25 double-positive cells were identified after gating for lymphocytes, with Foxp3-positive cells calculated among the gated cells.

### 2.6. Quantitative real-time reverse transcription-PCR

Total RNA was isolated from cells using Trizol reagent (Thermo Fisher Scientific), and complementary DNA was synthesized and used for qRT-PCR analysis. The qRT-PCR analysis was performed in 96-well plates with a LightCycler 480 (Roche Applied Science, Mannheim, Germany) using a SYBR Green I Master kit (Roche Diagnostics, Basel, Switzerland) according to the manufacturer’s protocols. The primers used for the PCR targeted the gene transcripts of the following proteins: lipoprotein lipase (*LPL*), glucose transporter type 4 (*GLUT4*), sex-determining region Y-box 9 (*SOX9*), collagen type 2 alpha 1 (*COL2A1*), collagen type 1 alpha 1 (*COL1A1*), runt-related transcription factor 2 (*RUNX2*), filaggrin, vascular endothelial growth factor (*VEGF*), interleukin (IL)-5, and β-actin. The thermocycling program used for amplification was as follows: pre-denaturation (95°C for 10 min) followed by 40 cycles of denaturation (95°C for 10 s), annealing (60°C for 10 s), and elongation (72°C for 10 s). Melting curve analysis was performed at 65–97°C to assess qRT-PCR product separation. The qRT-PCR results were calculated using Ct values. Relative quantification was conducted as previously described using β-actin as the reference gene. The 2^−ΔCT^ method described by Livak and Schmittgen was employed to normalize the gene expression values. The primer sequences and their respective annealing temperatures are listed in Table 2.

**Table 2:** List of primers used for qRT-PCR.

Genes	Primer sequence (5′–3′)	PCR product size (bp)	Annealing Tm (°C)
LPL	F: TTTGGGATACAGCCTTGGAG R: ACGACTTGGAGCTTCTGCAT	127	60
GLUT4	F: GCTTTGTGGCCTTCTTTGAG R: GTTGCTTGTCCAGTTGCAGA	125	60
SOX9	F: AGAAGGACCACCCGGACTAC R: CGTTCTTCACCGACTTCCTC	57	60
COL2A1	F: CTCAAGTCCCTCAACAACCAG R: TTGGGGTCGATCCAGTAGTC	134	60
COL1A1	F: AGAGGAGGGCCAAGAAGAAG R: AGATCACGTCATCGCACAAC	143	60
RUNX2	F: TCTGGCCTTCCACTCTCAGT R: GACTGGCGGGGTGTAAGTAA	161	60
Filaggrin	F: GATGACCCAGACACTGCTGA R: TGGTTTTGCTCTGATGCTTG	158	60
VEGF	F: GTAATGATGAGGGCCTAGAGTG R: TATGTGCTGGCCTTGATGAG	92	60
IL-5	F: CTTGGGGCTGCCTATGTTTC R: TCAGGTTCCCATCGCCTATC	118	60
β-actin	F: GCTACGTCGCCCTGGACTTC R: GCCCGTCGGGTAGTTCGTAG	86	60

### 2.7. Histopathological evaluation

Ear skin samples were collected, fixed in 10% formalin, subjected to consecutive tissue-processing steps including alcohol–xylene changes, and embedded in paraffin. Sections of 5 μm thickness were prepared and stained with hematoxylin and eosin.

### 2.8. Statistical analysis

The results are expressed as the mean ± SD for triplicate experiments (*n* = 3). Statistical significance was determined using GraphPad Prism 7 software (GraphPad Software, Inc., San Diego, CA, United States) with *t*-test or one-way ANOVA with Dunnett’s multiple comparisons test being used to compare groups. A value of *p* < 0.05 was considered statistically significant.

## 3. Results

### 3.1. cAT-MSC characterization and differentiation potential

**Figure 2:**
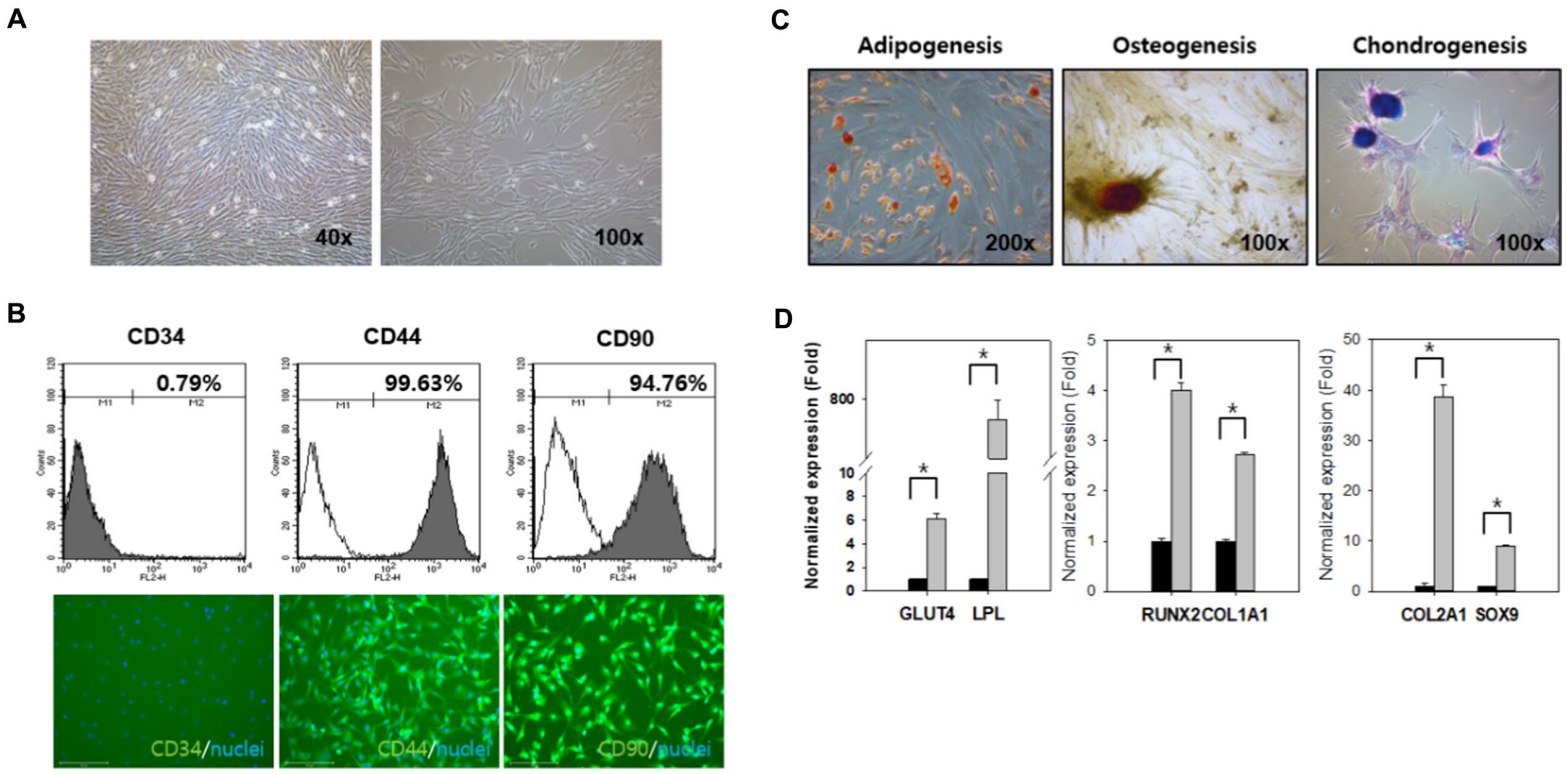
cAT-MSC isolation and characterization at Passage 3. **(A)** Cellular morphology showing a fibroblast-like shape (×40, ×100). **(B)** Flow cytometry histograms and immunocytochemistry (×100) showing the expression of selected markers (CD34, CD44, and CD90) in cAT-MSC populations compared to the control. Nuclei were stained with DAPI. **(C)** cAT-MSC differentiation potential in adipogenic, osteogenic, and chondrogenic media for up to 21 days were confirmed. Adipogenesis, osteogenesis, and chondrogenesis were demonstrated by Oil Red O, Alizarin Red, and Alcian Blue staining, respectively (adipogenesis = ×200, osteogenesis = ×100, and chondrogenesis = ×100). **(D)** Expression of differentiation markers in adipogenesis (*GLUT4* and *LPL*), osteogenesis (*RUNX2* and *COL1A1*), and chondrogenesis (*COL2A1* and *SOX9*) were evaluated using qRT-PCR. ^*^Significantly different between groups at *p* < 0.05 (Student’s *t*-test). cAT-MSC, canine adipose tissue-derived mesenchymal stem cell; qRT-PCR, quantitative reverse transcription PCR.

Canine adipose tissue-derived cells were able to both attach to the culture dishes and expand *in vitro* (Figure 2A). Flow cytometric analysis and immunocytochemistry revealed that third passage cells strongly expressed the cell-surface markers CD44 (99.63%) and CD90 (94.76%) and were negative for CD34 (0.79%; Figure 2B). To examine cAT-MSC mesodermal differentiation potential, the cells were differentiated into adipocytes, osteocytes, and chondrocytes under optimal conditions. Adipogenic differentiation was indicated by neutral lipid accumulation in the differentiated cells, as confirmed by Oil Red O staining (Figure 2C, left); osteogenic potential by the presence of extracellular calcium, as confirmed by Alizarin Red staining (Figure 2C, middle); and chondrogenic differentiation by strong blue proteoglycan staining (Figure 2C, right). The qRT-PCR revealed that the expression of adipogenic (*GLUT4* and *LPL*), osteogenic (*RUNX2* and *COL1A1*), and chondrogenic differentiation potential-related genes (*COL2A1* and *SOX9*) was significantly higher (*p* < 0.05) in the differentiated cells than in the undifferentiated cells (Figure 2D, left, middle, and right, respectively). These results indicate the stem cell plasticity of canine adipose tissue-derived cells.

### 3.2. cAT-MSC effects on immune responses in AD

**Figure 3:**
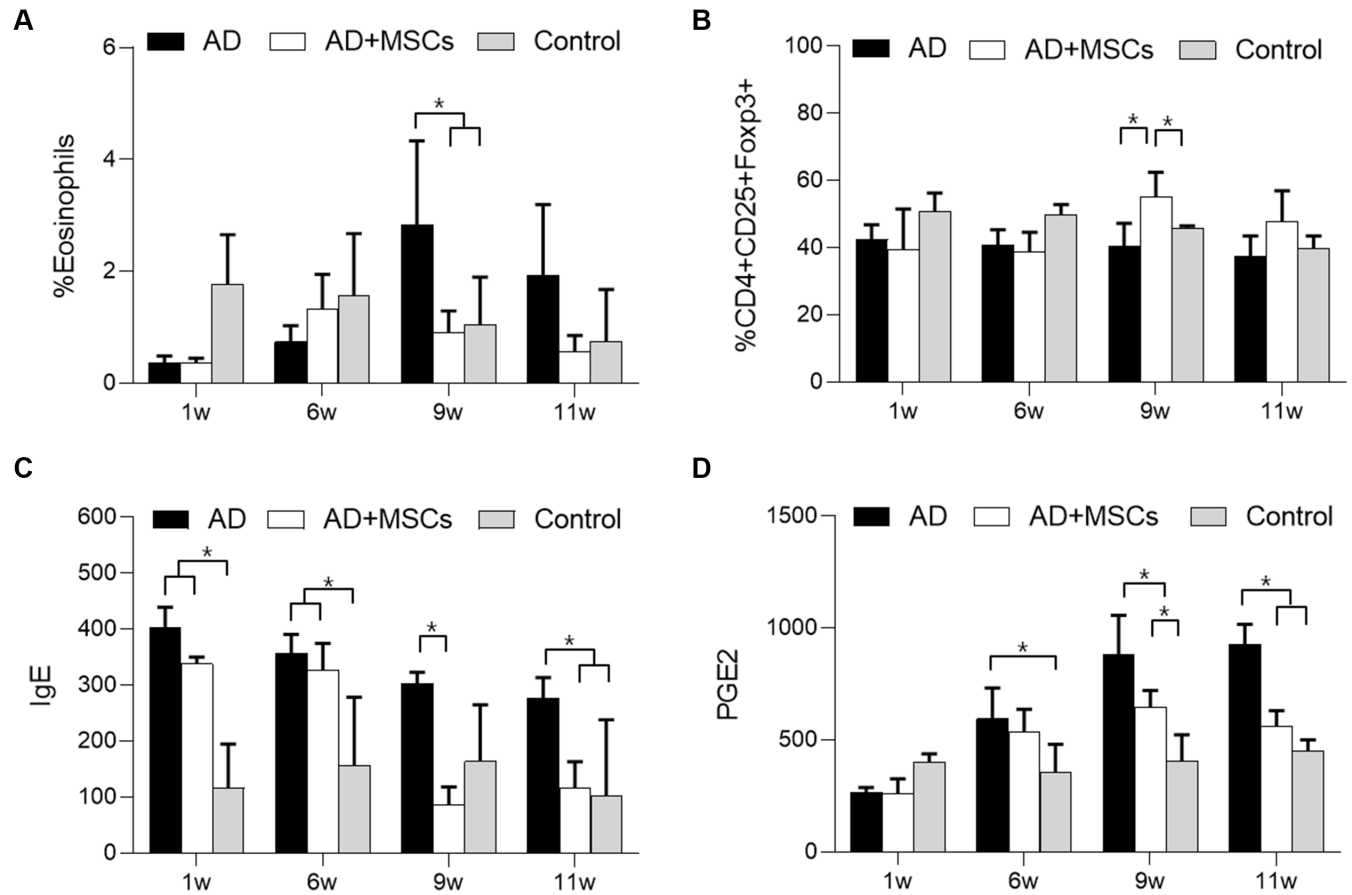
Effects of cAT-MSCs on immune responses in atopic dermatitis. The ratio of **(A)** eosinophils and **(B)** Tregs in PBMCs as determined by FACS analysis and **(C)** IgE and **(D)** PGE2 concentrations were measured over 11 weeks in the AD, AD + MSCs, and control groups. ^*^Statistically significant difference among groups at *p* < 0.05 (Dunnett’s test). AD, atopic dermatitis; MSC, mesenchymal stem cell; cAT-MSC, canine adipose tissue-derived mesenchymal stem cell; FACS, fluorescence-activated cell sorting; IgE, immunoglobulin E; PBMC, peripheral mononuclear blood cell; PGE2, prostaglandin E_2_; and Treg, regulatory T cell.

We investigated whether immunomodulatory factors were altered in the control, AD, and AD + MSCs groups. Prior to cAT-MSC administration at week 6, the number of eosinophils following DFE treatment was lower in the two AD-induced groups than in the control group, although the difference was not significant. The number of eosinophils at week 9 increased significantly (*p* < 0.05) in the AD group but not in the AD + MSCs group; this level was similar to that of the control (Figure 3A). Regarding the change in Treg (CD4^+^CD25^+^Foxp3^+^) numbers among the PBMCs, the ratio prior to the 6-week mark was lowered in the two AD-induced groups compared with that of the control group. The Tregs ratio significantly increased (*p* < 0.05) at week 9 in the AD + MSCs group but not in the AD group (Figure 3B; Supplementary Figures S1–S3). Prior to the 6-week mark, IgE levels were higher in the serum of dogs treated with DFE (AD and AD + MSCs groups) than in the control group. After 9 weeks, IgE levels decreased in the AD + MSCs group (Figure 3C); however, they were still higher in the AD group than in the control and AD + MSCs groups. PGE2 levels were significantly higher (*p* < 0.05) in the AD group than in the control group; however, after 9 weeks, they were significantly lower (*p* < 0.05) in the AD + MSCs group than in the AD group (Figure 3D).

### 3.3. cAT-MSC effects on AD skin lesions

**Figure 4:**
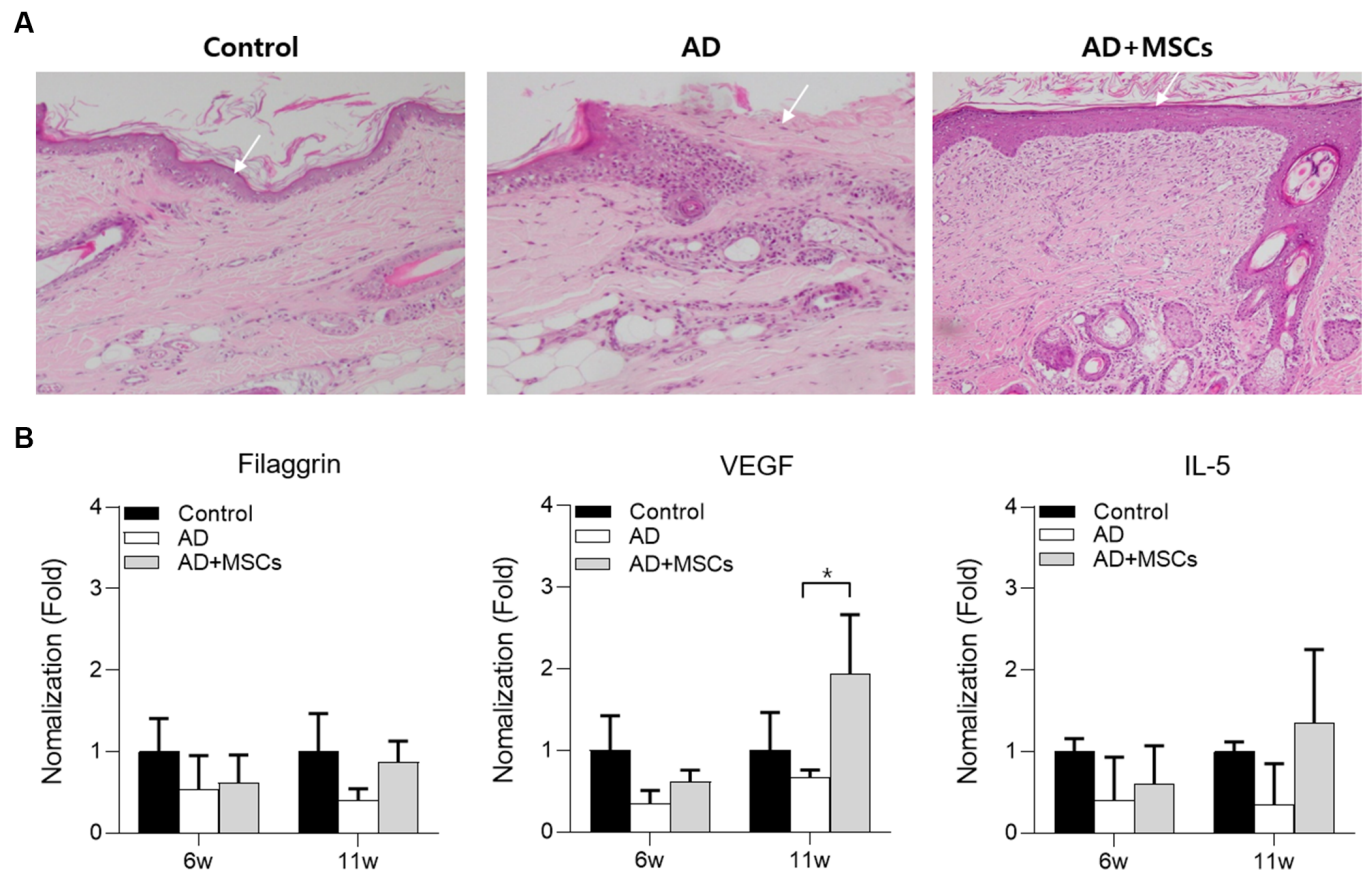
Histological analysis of ear skin tissue after 12 weeks. **(A)** Ear skin tissue of dogs with control, AD, or AD induced by subcutaneous injection were stained with H&E for cAT-MSCs. **(B)** The serum filaggrin, VEGF, and IL-5 expression levels in AD and AD + MSCs groups were relatively quantified against those of the control at 6 and 11 weeks. ^*^Statistically significant differences among groups at *p* < 0.05 (Dunnett’s test). AD, atopic dermatitis; cAT-MSC, canine adipose tissue-derived mesenchymal stem cell; IL, interleukin; H&E, hematoxylin and eosin; MSC, mesenchymal stem cell; and VEGF, vascular endothelial growth factor.

We observed a clinical improvement after cAT-MSCs treatment; the severity of week 11 ear skin lesions was reduced more than those of week 6 in the AD + MSCs group compared to AD group (Supplementary Figure S4). Histologically, the AD group showed epidermal loss and inflammatory cell infiltration (Figure 4; Supplementary Figure S5). The AD + MSCs group also showed infiltrating inflammatory cells, though these appeared to be partially replaced by normal tissue cells as shown by high fibroblast infiltration around the epidermis and collagen fiber maturation (Figure 4; Supplementary Figure S6).

Thereafter, the expression levels of filaggrin, VEGF, and IL-5 were examined in the control, AD, and AD + MSCs groups following cAT-MSC treatment at week 6. To compare gene expression among groups, the two AD-induced group expression levels were normalized against those of the control group at weeks 6 and 11. The expression levels of all genes were relatively lower in the two AD-induced groups at week 6 compared with those of the control group. However, the expression of all genes was increased at the 11-week mark in the AD + MSCs group compared with that in the AD group (Figure 4B).

## 4. Discussion

Atopic dermatitis is a widespread chronic inflammatory skin disease associated with allergies and can be induced by house dust-mite inhalation as an airborne allergen. Various AD models have been developed in mice (28, 29) and dogs (30, 31). Marsella et al. (29) reported that a canine house dust mite-induced AD model showed high IgE production, severe cutaneous lesions, and pruritus after infection. Additionally, Olivry et al. demonstrated that experimental canine house dust mite-induced AD skin lesions exhibited active innate and adaptive immune responses and pruritogenic pathways, similar to those of patients with acute atopic symptoms (12). They also observed that acute canine AD skin lesions exhibited substantial upregulation of genes encoding cytokines related to Th2 cells (e.g., IL-4, IL-5, IL-13, IL-31, and IL-33), Th9 cells (IL-9), and Th22 cells (IL-22) as well as the Th2-promoting chemokines, chemokine ligand (CCL)5 and CCL17 (31).

The AD model was established with DFE ointment in this study. *Dermatophagoides farinae* is a type of American house dust-mite and the extract is a causative allergen that induces AD in dogs (30, 32). Topical application of DFE is known to induce AD-like skin lesions in experimental mice; clinical manifestation begins with skin dryness and is followed by mild erythema, hemorrhage, and edema (33). Ultimately, the skin becomes severely thickened and erythematous; hemorrhage, edema, scarring, erosion, and excoriation are also observed (34). In this study, we found that repeated application of DFE ointment induced AD-like skin lesions in dogs. Previous studies on AD have suggested that skin lesions carry topical eosinophilia, cause systemic IgE elevation, and are associated with Th2-type cytokine expression (28). Accordingly, serum IgE levels were elevated in the DFE-induced AD group compared to those in the control group; the number of eosinophils also increased in the AD group. Histologically, the skin lesions of the subjects with AD exhibited significant dermis and epidermis thickening as well as hyperkeratosis and parakeratosis.

An increased number of dermal inflammatory cells including mast cells, eosinophils, and lymphocytes was observed in the lesions. This was not surprising as eosinophilic infiltration of AD lesions, along with increased eosinophil numbers in the peripheral blood, is frequently seen in patients with AD. In addition, eosinophils serve as FcεRI-expressing effector cells in IgE-associated allergic inflammation (35). In contrast to the observation of inflammatory cell infiltration in the AD lesion, no eosinophil increase was discerned until week 6 of AD-induction. Lower numbers of eosinophils were counted in the DFE-treated groups (AD and AD + MSCs) compared with those in the control group; however, the difference was not significant. Eosinophil numbers and their granule protein levels are elevated in most patients with AD and they are correlated with disease severity (36). We concluded that eosinophil number, which was not elevated during DFE treatment, might be a result of mild AD severity. However, further studies on whether eosinophil count in peripheral blood is an appropriate AD indicator are needed.

The roles of Th1 and Th2 cytokines in the skin inflammatory response have been demonstrated by the targeted regulation (overexpression or deletion) of these cytokines in allergen-induced experimental mouse models. Skin-localized expression of Th2 cytokines may play a decisive role in AD development, as suggested by the finding that transgenic mice that overexpress these cytokines in their skin develop inflammatory and pruritic skin lesions similar to AD (37). Allergen-sensitized skin from IL-5-knockout mice exhibits decreased thickening and no detectable eosinophils. The skin from IL-4-knockout mice exhibits normal thickening but reduced eosinophil numbers, whereas the skin of interferon-γ-knockout mice is characterized by reduced dermal thickening (38). IL-5 is a glycoprotein mainly produced by Th2 cells, activated eosinophils, and mast cells and it promotes eosinophilic precursor maturation both within and outside the bone marrow (39, 40). The cytokine milieu in the skin exerts modulatory effects on filaggrin expression, and Th2 cytokines negatively modulate filaggrin expression in keratinocytes (41). In this study, the IL-5 and filaggrin expressions were not elevated by cAT-MSC treatment in the AD + MSCs group. VEGF is a powerful stimulating factor for angiogenesis and vascular permeability of the skin (42). The VEGF cellular sources are endothelial cells, fibroblasts, smooth muscle cells, and macrophages. VEGF is involved in different stages of angiogenesis, including vasodilatation, endothelial cell permeability, perivascular matrix remodeling via induction of metalloprotease I and plasminogen activators, and induction of endothelial cell proliferation and migration. VEGF is also a potentially important mediator in contact eczema pathogenesis associated with delayed hypersensitivity (43). In this study, VEGF expression significantly increased in the AD + MSCs group, thereby demonstrating that MSCs induce cytokine modulation in the canine AD model.

The balance between Th1 and Th2 responses is important for determining skin health when exposed to certain allergens. Tregs have immunoregulatory functions and a cytokine profile distinct from that of either Th1 or Th2 cells (44). They represent up to 10% of all CD4^+^ T cells and further divide into groups including naturally occurring thymus-derived and peripherally-induced Tregs (5, 6). Tregs are important immune homeostasis regulators and play an essential role in human AD, with many studies having reported altered numbers of circulating Tregs in patients with AD compared with those in healthy humans (9, 11). Given the similarities in human and canine immune systems, it is likely that Tregs also play an essential role in canine AD; however, very little is known about such a role. In the current study, we observed that the number of CD4^+^CD25^+^ Tregs among PBMCs was lower in the DFE-induced AD group than in the control group; however, this ratio was significantly elevated after treatment with cAT-MSCs. Thus, MSCs may play an important immunomodulatory role in canine AD models.

Atopic dermatitis is also characterized by dry skin, which comprises non-lesioned skin and increased transepidermal water loss. This skin barrier dysfunction leads to increased antigen absorption, contributing to characteristic AD cutaneous hyperreactivity (45). Filaggrin is an epidermal filament-aggregating protein that has an essential structural and functional role in the epidermis and can affect skin homeostasis. Filaggrin deficiency has been linked to AD pathogenesis. Pendaries et al. (46) showed that filaggrin deficiency in a fibroblast- and immune cell-free reconstructed human epidermis resulted in some of the epidermal alterations observed in patients with AD. They further showed that filaggrin deficiency resulted in reduced keratohyalin granules and epidermal thickness (46).

Inflammation is a defensive response to damaging external stimuli and it initiates tissue repair and remodeling; however, when dysregulated, it can have harmful effects. MSCs can be used as alternative cell therapies for the treatment of several immune disorders. Specifically, MSC therapy offers an attractive substitute for immune disorder treatments, including those of relapsing or refractory types (47). The effects of MSCs in AD are well reviewed by Kim et al. (48); Th2 polarization shift, decrease in IgE levels, allergen-specific Tregs elevation, Th2 cytokine downregulation, and eosinophil level reduction. Similar to reported immunomodulatory MSC effects, we successfully demonstrated that cAT-MSCs reduced eosinophil, IgE, and PEG2 levels, and elevated the percentage of Tregs in an experimental canine DFE ointment-induced AD model. Furthermore, filaggrin, VEGF, and IL-5 expression was increased by MSC treatment, however, not significantly. Nevertheless, limitations remain that need to be addressed in the future, such as bias due to individual differences and the small number of experimental animals.

## Data availability statement

The original contributions presented in the study are included in the article/Supplementary material; further inquiries can be directed to the corresponding authors.

## Ethics statement

The animal study was reviewed and approved by Korean animal medical science institute (KAMSI), the guidelines of the Institute of Laboratory Animal Resources (15-KE-047).

## Author contributions

S-JK, JL, and D-KY: conceptualization. N-YG and JB: methodology. S-JK and JL: writing the manuscript. B-HH, JL, and D-KY: discussion. All authors contributed to the article and approved the submitted version.

## Funding

This work was funded by the Animal and Plant Quarantine Agency grant number (M-1543083-2014-15-02 and N-1543083-2023-32-01), Republic of Korea.

## Conflict of interest

The authors declare that the research was conducted in the absence of any commercial or financial relationships that could be construed as a potential conflict of interest.

## Publisher’s note

All claims expressed in this article are solely those of the authors and do not necessarily represent those of their affiliated organizations, or those of the publisher, the editors and the reviewers. Any product that may be evaluated in this article, or claim that may be made by its manufacturer, is not guaranteed or endorsed by the publisher.
