# Supplementary material for: Immunomodulatory effects of canine mesenchymal stem cells in an experimental atopic dermatitis model
Source: Front Vet Sci. 2023 Jul 13;10:1201382. doi: 10.3389/fvets.2023.1201382 (PMC10390254; doi:10.3389/fvets.2023.1201382)

**Figure S1.** Dot plot of Tregs using flow cytometry at 1, 6, 9 and 11 wk of control group (n=3).


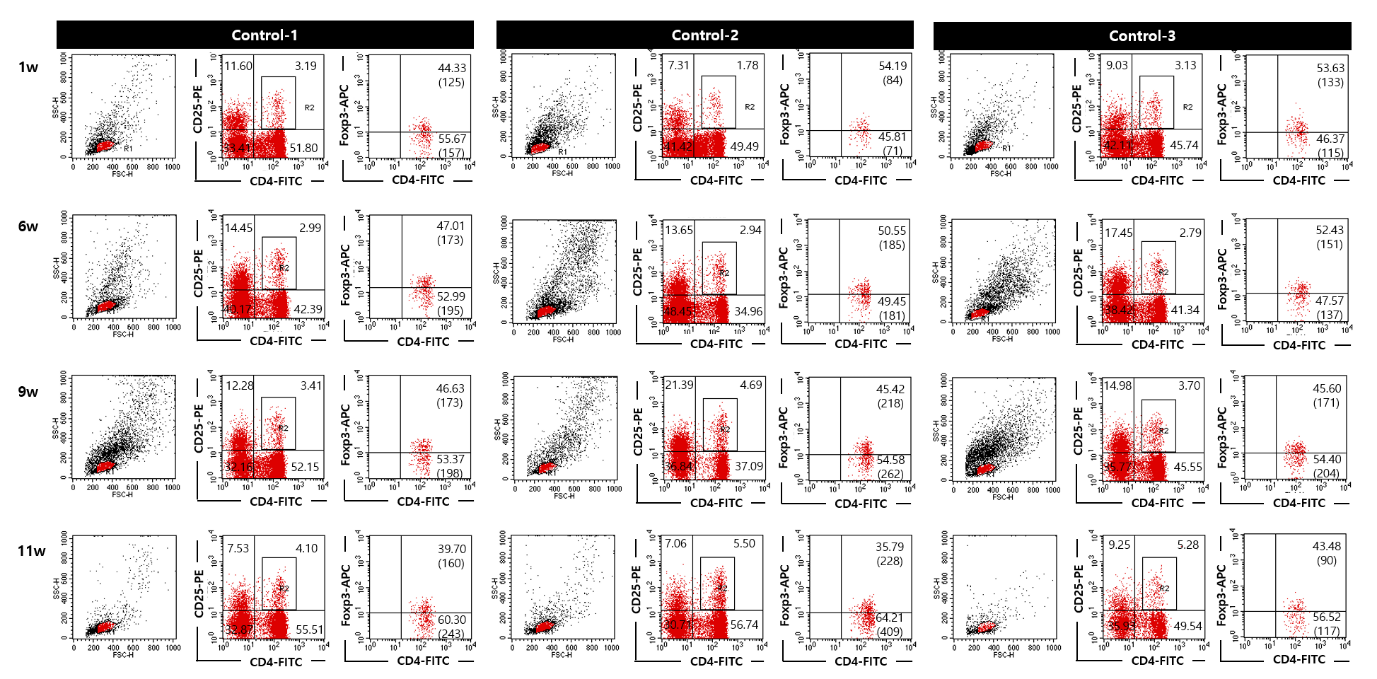


**Figure S2.** Dot plot of Tregs using flow cytometry at 1, 6, 9 and 11wk of AD group (n=3).


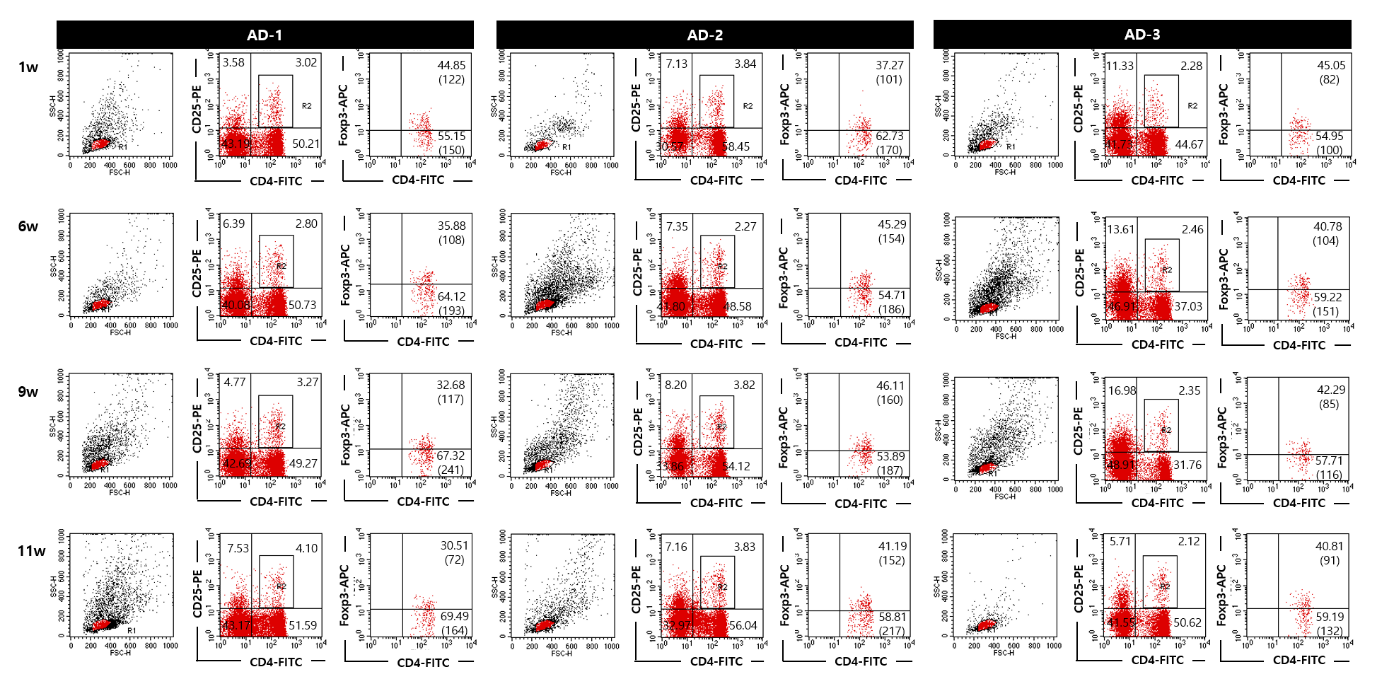


**Figure S3.** Dot plot of Tregs using flow cytometry at 1, 6, 9 and 11wk of AD+MSCs group (n=3).


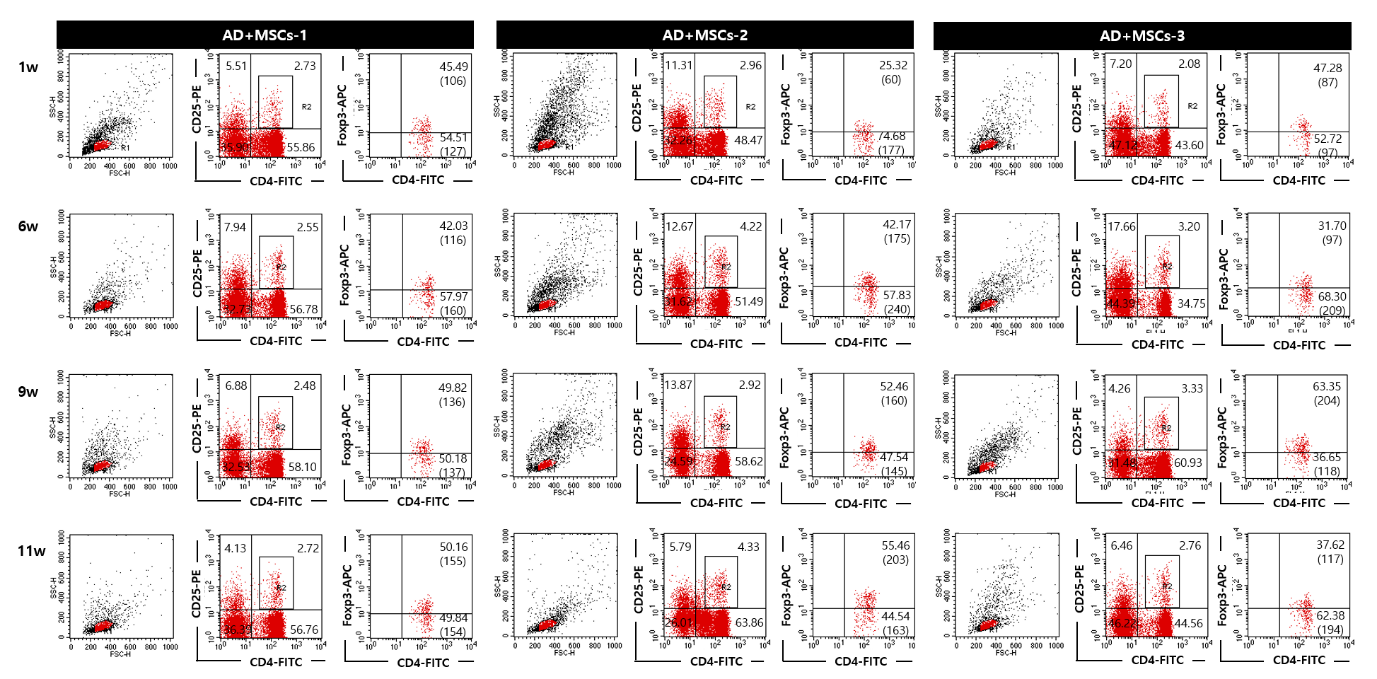


**Figure S4.** Dog ears of pre- (6 wk) and post-treatment (11 wk) of cAT-MSCs.


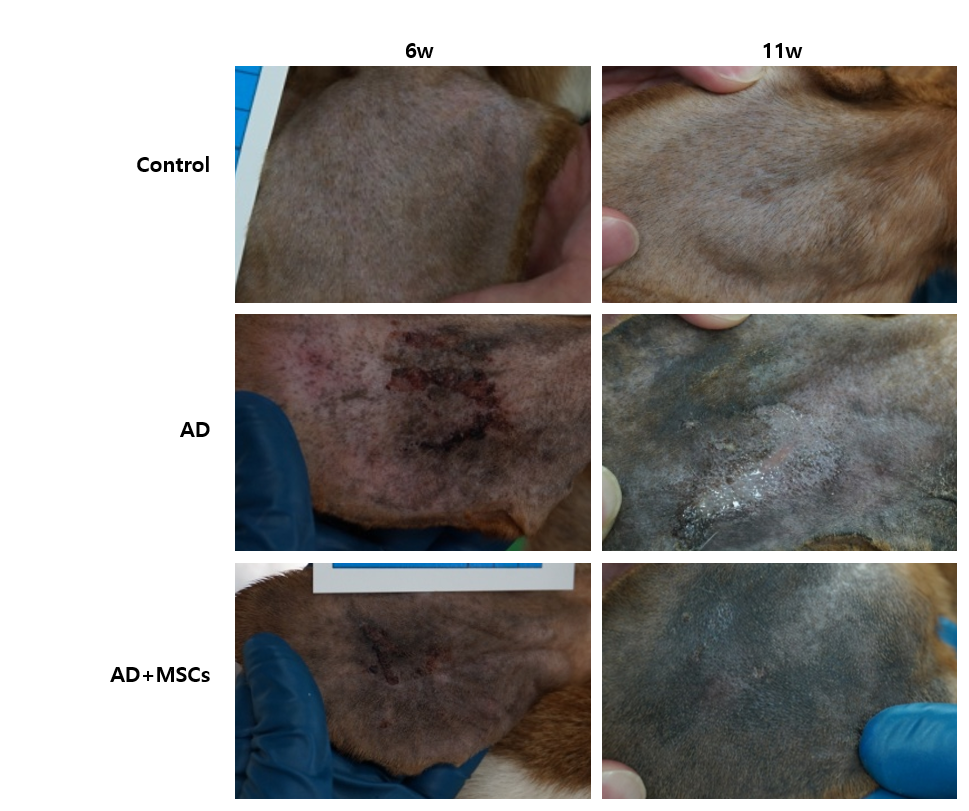


**Figure S5.** Histological analysis of ear skin tissue of dog in AD group.


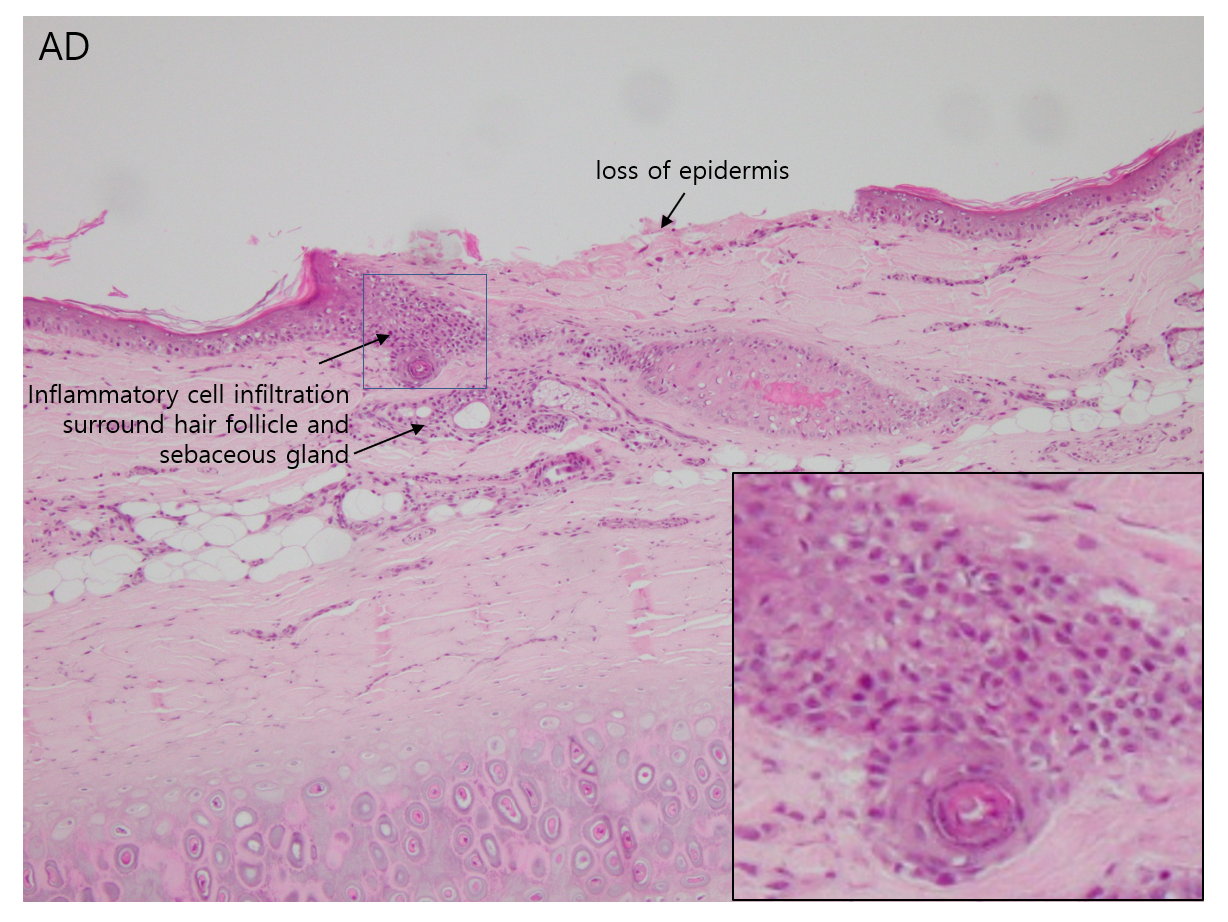


**Figure S6.** Histological analysis of ear skin tissue of dog in AD+MSCs group.


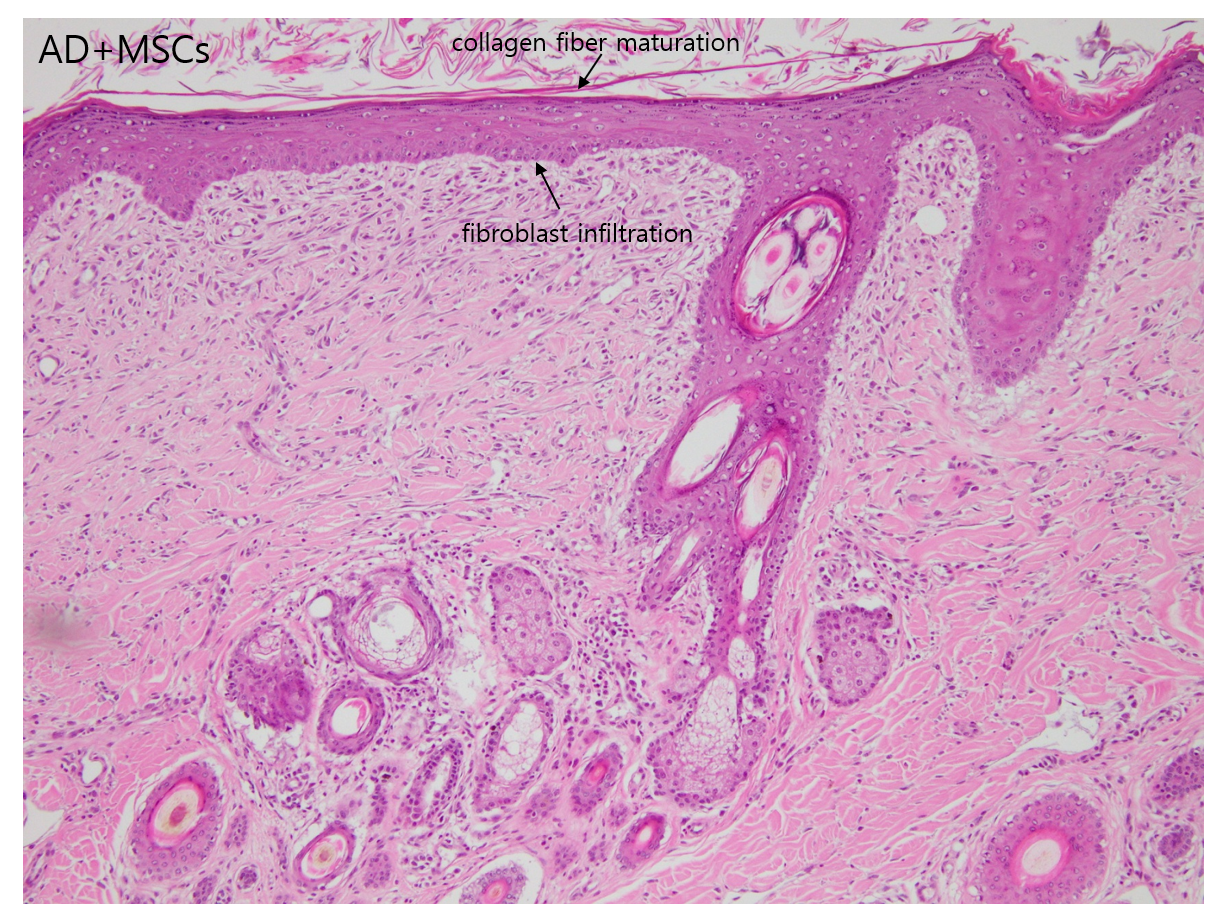

Supplement: Supplementary file 1 [file Data_Sheet_1.docx]
